# The Evolving Treatment Landscape for Metastatic Differentiated Thyroid Cancer

**DOI:** 10.6004/jadpro.2014.5.6.7

**Published:** 2014-11-01

**Authors:** Carolyn Grande, Marcia S. Brose

**Affiliations:** Hospital of the University of Pennsylvania, Abramson Cancer Center, Philadelphia, Pennsylvania

Review of "*Sorafenib in metastatic thyroid cancer: A systematic review*" by Thomas et al. (2014), The Oncologist, 19, 251–258. *For a discussion of meta-analysis and forest plots used in oncology research, please see the related article by Joanne Lester starting on page 465.*

Nearly 63,000 new cases of thyroid cancer will be diagnosed in 2014, with an estimated death rate of 1,900 during this period. Differentiated thyroid cancer (DTC) includes papillary, follicular and Hürthle cell histologies and accounts for greater than 90% of all thyroid cancers (American Cancer Society, 2014).

While most differentiated thyroid cancers are curable with implementation of the standard of care including surgery, possible radioactive iodine (RAI), and thyroid stimulating hormone (TSH) suppression, 10% to 15% of patients will have or develop disease that is RAI-refractory or nonavid (Pacini et al., 2012). This population of patients has a poorer prognosis with an overall survival rate of 2.5 to 3.5 years (Durante et al., 2006).

For patients who are refractory to RAI and have metastatic or recurrent disease not amenable to surgery or external-beam radiation therapy, there has been a paucity of response to traditional chemotherapeutic agents. In this setting, cytotoxic agents such as doxorubicin have produced insignificant improvement in objective and subjective sequelae or overall survival benefit (Gottlieb et al., 1974).

The shift in systemic oncologic treatment from chemotherapeutic agents to targeted agents has been practice-changing in a variety of hematologic and solid tumor malignancies. The advent of multikinase inhibitors (MKIs) that have the ability to target a variety of overexpressed mutated pathways and block angiogenesis signaling has been promising.

Thyroid cancer, recognized as highly vascular, also has multiple associated somatic mutations of proto-oncogenes v-Raf murine sarcoma viral oncogene homolog B (BRAF), V-Ki-ras2 Kirsten rat sarcoma viral oncogene homolog (K-Ras), and rearranged during transfection (RET).

These mutations, among others, are responsible for the progression of an estimated 70% of thyroid carcinomas (Nikiforov et al., 2011). Given the array of potential molecular targets in RAI-refractory DTC, the role of MKIs has been studied in a plethora of phase II and III clinical trials. The intent of this article is to provide a perspective on the meta-analysis of sorafenib (Nexavar) use in phase II clinical trials for treatment of patients with metastatic thyroid cancer recently published in *The Oncologist* by Thomas and colleagues (2014). Additionally, we will discuss the authors’ conclusions as they relate to those achieved in the pivotal phase III trial (Brose et al., 2014) that garnered the United States Food and Drug Administration (FDA) approval of sorafenib in the treatment of this patient population.

## LITERATURE REVIEW

Thomas and colleagues (2014) performed a systematic review of the literature for use of sorafenib in the treatment of metastatic thyroid cancer. Goals of the review were to assess the efficacy of sorafenib in this setting and perform a meta-analysis of response rates, median progression-free survival, and the incidence of adverse events associated with treatment. The search returned nine studies, two of which were excluded because they did not meet inclusion criteria and one of which was excluded because of different drug dosing. Five studies were phase II and two studies were retrospective analyses. A total of 219 patients with metastatic thyroid cancer were included in the review. Histologically, 159 patients had DTC, 52 had medullary thyroid cancer (MTC), and 8 had anaplastic thyroid cancer (ATC).

The overall partial response (PR) rate was 21% for DTC, 22% for MTC, and 13% for ATC. The overall clinical benefit (PR and stable disease responses) was 79% for DTC and 93% for MTC. Overall survival (OS) was not reported in four of the six articles. The remainder of the studies reported OS at 100% at 2 years (Ahmed et al., 2011), 23.6 months as median OS (Capdevila et al., 2012), and a median OS of at least 23 months, with papillary thyroid cancer patients who had received prior chemotherapy achieving an OS of 37.5 months (Kloos et al., 2009). Sixteen percent of patients discontinued medication because of adverse events (AEs), and 56% had dose reductions for toxicity. The most common dose reduction was from 800 mg total daily dose to 400 mg once daily. Adverse events are summarized in the Table. The authors concluded that sorafenib was a promising treatment option in patients with progressive DTC and MTC, yet they advised careful patient selection due to the high rate of AEs requiring dose reduction as well as careful management.

**Table 1:**
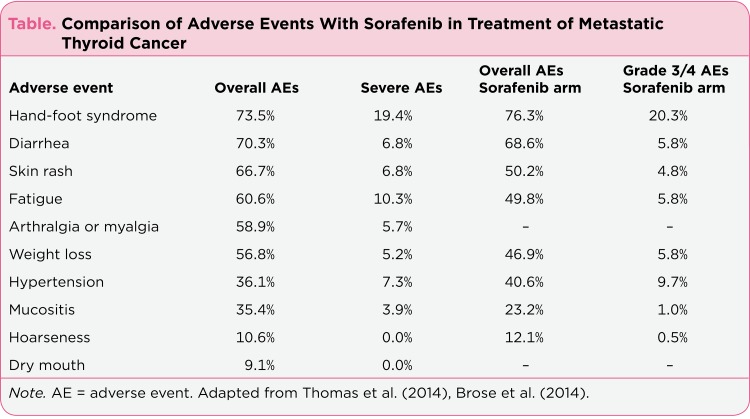
Comparison of Adverse Events With Sorafenib in Treatment of Metastatic Thyroid Cancer

## DECISION TRIAL

At the time of their publication (Thomas et al., 2014), the data from the phase III, multicenter, randomized, double-blind, placebo-controlled trial (DECISION) in radioactive iodine-refractory, locally advanced or metastatic DTC had been reported at the American Society of Clinical Oncology annual meeting but not yet published. In this trial, patients were randomly assigned on a 1:1 basis to either sorafenib or placebo. There were 417 patients in the intent-to-treat population: 207 in the sorafenib group and 210 in the placebo group. There were 416 patients in the safety population: 207 in the sorafenib group and 209 in the placebo group. The DECISION trial reported that sorafenib significantly improved the median progression-free survival for patients randomized to sorafenib at 10.8 months compared to those on placebo at 5.8 months, with a 41% reduction in the risk of progression or death during the double-blind period (Brose et al., 2014).

Sorafenib is the first MKI approved for treatment of locally recurrent or metastatic progressive DTC refractory to RAI treatment. This November 2013 approval was based on completion of the first phase III study for this indication (Brose et al., 2014). Sorafenib is an inhibitor of RET, VEGFR1, VEGFR2, VEGFR3, Flt3, c-KIT, and wild type and mutant (V600E) BRAF (Bayer, 2013).

## DRAWING CONCLUSIONS CAREFULLY

In academia, it is prudent and advisable not to perform a direct trial-to-trial comparison in terms of efficacy results. With that in mind, particular focus on the cautionary conclusions made by the authors of the systematic review as they relate the high AE rate and dose reductions therein should be addressed. As summarized in the Table, the adverse events experienced by metastatic thyroid cancer patients being treated with sorafenib were typical of those reported in the sorafenib safety profile. Dose interruptions, reductions, or withdrawals because of AEs in the DECISION trial occurred at 66.2%, 64.3%, and 18.8%, respectively. Despite the greater than 60% dose interruptions or reductions, the trial met its primary endpoint.

The DECISION trial established that a dose reduction to 600 mg total daily dose was a reasonable approach for most toxicities. This was not the standard reduction in any of the referenced papers in the meta-analysis, with the exception of Gupta-Abramson and colleagues (2008). While Thomas and colleagues note that 16% of patients discontinued due to toxicity, it is most important to know when this occurred. When toxicity is well controlled, patients may be treated for extended periods (over 18 months) but subsequently decide to discontinue treatment due to toxicity. These patients are likely to have already received benefit from treatment, in spite of ultimately discontinuing for toxicity. Where the authors note that 16% discontinued due to toxicity and an additional 56% had reduced doses, it is likely that this is an overlapping group of patients, thus the authors assume a higher rate of dose adjustment due to severe AEs.

## ADVERSE EVENTS

The inclusion of two retrospective reviews in the meta-analysis further muddies the waters, as dose reduction for toxicity will not have followed any guidelines and thus are not comparable for adverse events and should have been removed from the analysis.

Critical in this patient population is identifying that the majority of the AEs experienced are predictable and time-limited. Hypertension has been reported to occur in a higher incidence in patients with DTC on sorafenib therapy. Hypertension usually occurs in the first 6 weeks of therapy, thus close monitoring of patient’s blood pressure and early intervention can minimize dose interruptions or reductions.

A macular or papular rash on the face, neck, upper chest, back, and extremities is a common occurrence in patients being treated with sorafenib. In a phase II trial of metastatic thyroid cancer patients being treated with sorafenib, a grade 2/3 rash peaked at cycle 1 (19%) and declined by cycle 3 (5%; Terry et al., 2013).

Hand-foot skin reaction (HFSR) was the most common AE in the meta-analysis and in the DECISION trial; however, in DECISION, only 11 patients discontinued treatment related to this reaction. In a phase II trial of metastatic thyroid cancer patients being treated with sorafenib, grade 2/3 HFSR peaked at cycle 2 (39%) and decreased by cycle 6 (10%), with 31% of patients requiring dose reduction (Terry et al., 2013). Proper preventative techniques and management beginning with the first signs of HFSR can sustain patients through the anticipated time frame in order to continue therapy without dose interruptions or reductions (Lacouture et al., 2008).

Fatigue for patients with DTC in a phase II clinical trial commonly occurred in the first 4 to 6 months of treatment, often resolving following 5 to 6 months of treatment. Fatigue is typically multifactorial in origin and self-limiting. It generally does not require dose adjustments of sorafenib in DTC patients.

For patients with DTC being treated with sorafenib, diarrhea onset may be slow, occurring up to 6 months after initiation of therapy. This can be aggravated by lifestyle factors including dietary selection. Diarrhea was noted in the systematic review to occur at 70.3% in all grades and 6.8% in severe grades; this was slightly higher than in the DECISION trial at 68.6% and 5.8%, respectively. It could be that the rates were slightly higher due to the inclusion of patients with MTC in the systematic review, as these patients are prone to diarrhea as sequelae of increased calcitonin and not a treatment effect. For patients with DTC treated with sorafenib, it has been observed that diarrhea is episodic, occurring intermittently between 2 to 3 days a week in the majority of patients. The need to dose-reduce or interrupt sorafenib therapy for grade 1 or 2 diarrhea is rare (Brose et al., 2014).

## CONCLUSIONS

The approval of sorafenib in the treatment of patients with metastatic differentiated thyroid cancer serves as a practice-changing shift in management of this population. The safety profile is well documented, along with the onset and duration of common AEs in these patients. In addition to clear evidence of efficacy in this disease, the DECISION trial presents a comprehensive approach to management of adverse events, including the use of a brief dose interruption and initial dose reductions to a total daily dose of 600 mg a day.

Medical oncology health-care providers seasoned in assessment, treatment, and management of these patients can assist in minimizing the severity and duration of AEs while achieving desired efficacious outcomes. Resources are available for the medical oncology practitioner not experienced in the use of sorafenib or treatment of thyroid cancer patients (Brose et al., 2014; Walko & Grande, 2014). Being experienced and astute in this aspect of care can demystify the patient selection for this novel therapy in the metastatic thyroid cancer setting, particularly given that it is a disease that finally has received a long-awaited treatment option, where no others existed.

## FUTURE DIRECTIONS

The absence of evidence-based data to appropriately prevent or manage sorafenib treatment-related side effects is desperately needed. Many unanswered questions remain with regard to the dermatologic and gastrointestinal AEs. This presents an opportunity for development of adjunct research in these patients in an effort to preserve quality-of-life and therapeutic outcomes.
